# Supplementary material for: FAM9B serves as a novel meiosis-related protein localized in meiotic chromosome cores and is associated with human gametogenesis
Source: PLoS One. 2021 Sep 10;16(9):e0257248. doi: 10.1371/journal.pone.0257248 (PMC8432983; doi:10.1371/journal.pone.0257248)
Supplement: S1 Raw images — (PDF) [file pone.0257248.s001.pdf]

FAM9B expression was determined by qPCR, RT-PCR, and western blotting.

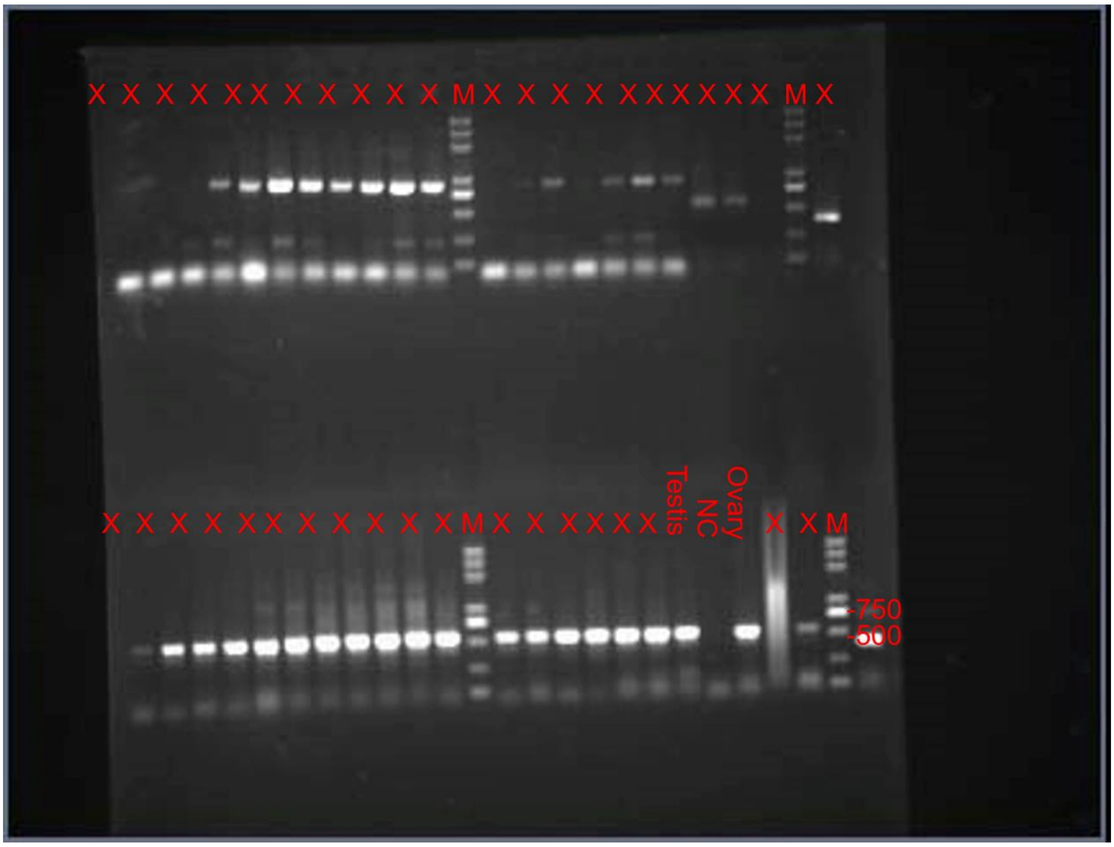

(A) qPCR results show that FAM9B mRNAs are highly expressed in testes and ovarian tissue.

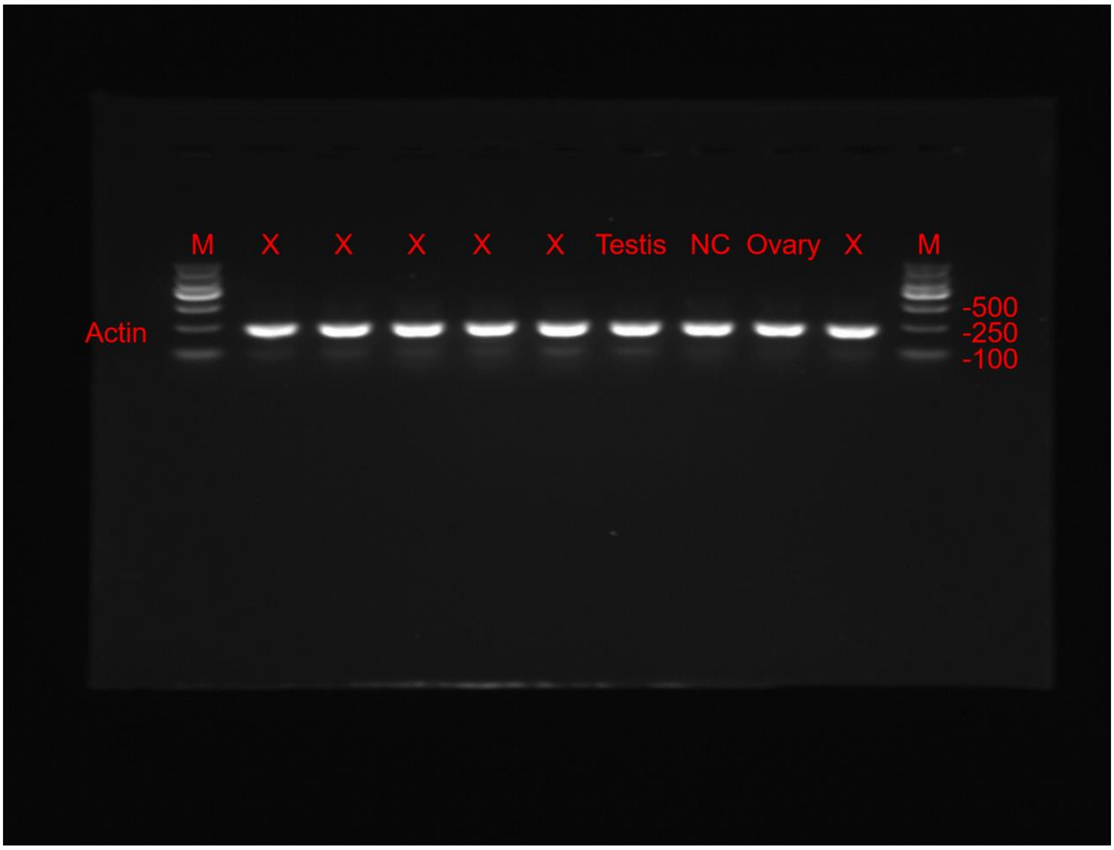

(B) qPCR results show that Actin mRNAs are highly expressed in testes and ovarian tissue.

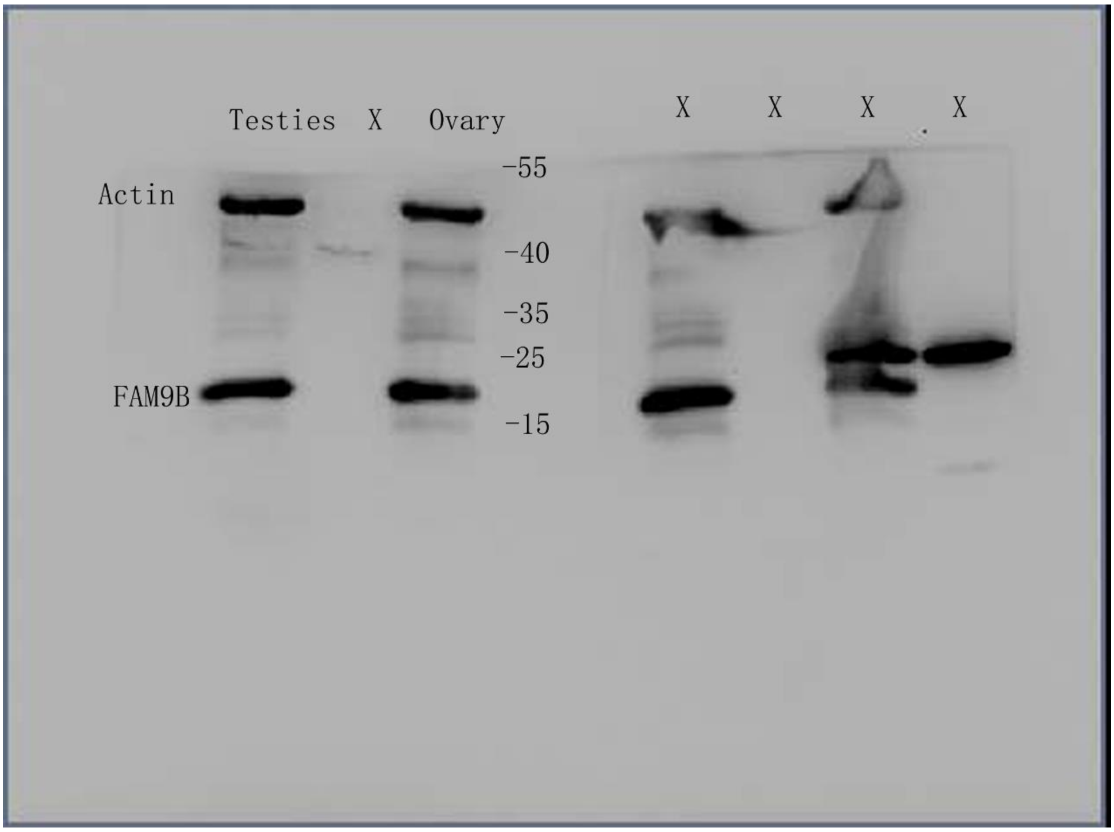

(C) Western blotting results for FAM9B expression in both testes and ovaries. Actin protein were used as internal control. All experiments were repeated and analyzed at least three times. All bar graphs show the mean±s.e.m. *P<0.05, **P<0.001.
