# Supplementary material for: FAM9B serves as a novel meiosis-related protein localized in meiotic chromosome cores and is associated with human gametogenesis
Source: PLoS One. 2021 Sep 10;16(9):e0257248. doi: 10.1371/journal.pone.0257248 (PMC8432983; doi:10.1371/journal.pone.0257248)
Supplement: S2 Raw images — (PDF) [file pone.0257248.s002.pdf]

Immunostaining results for FAM9B expression in human testicular sections.

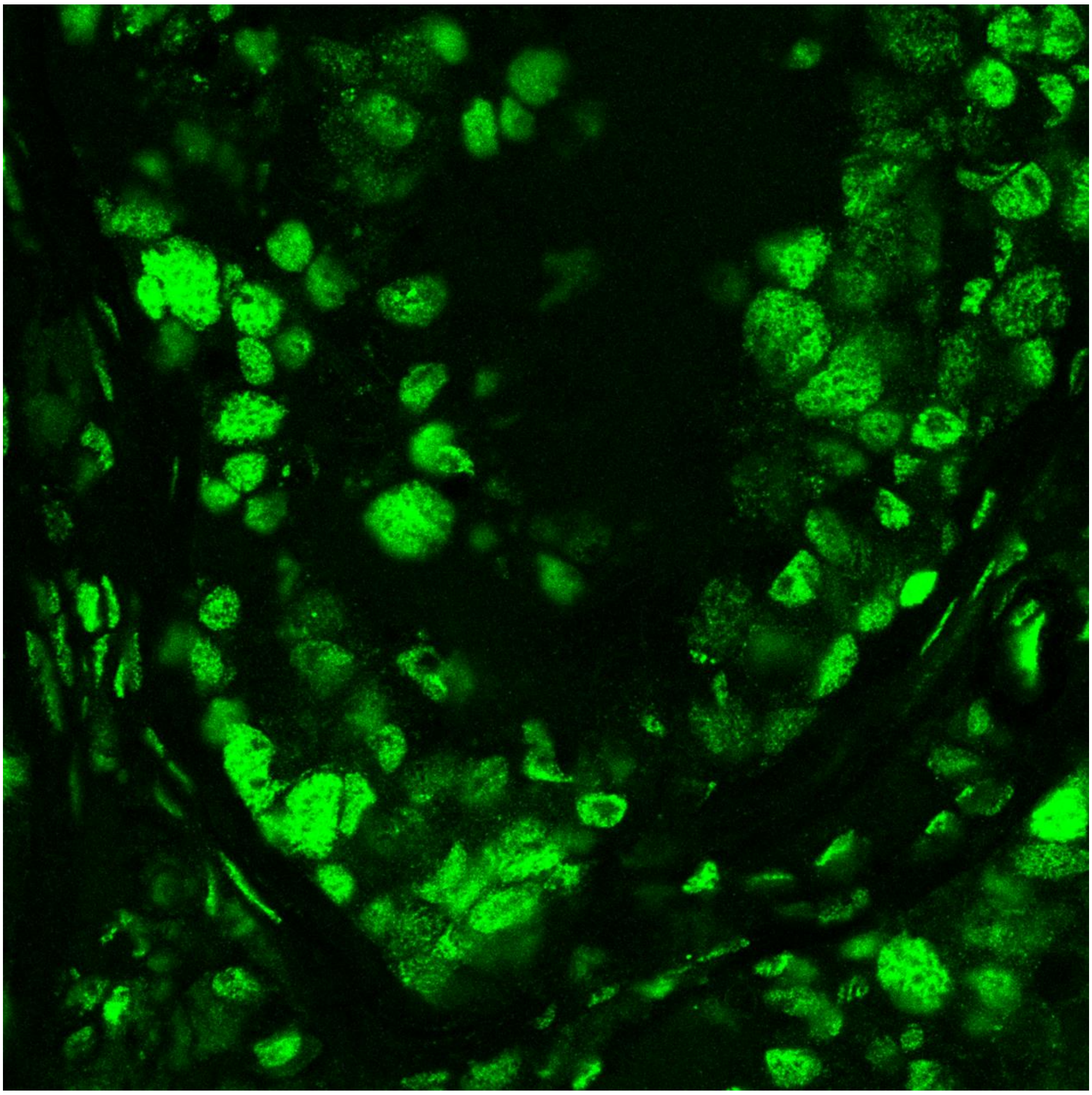

(A) FAM9B (green) is clearly present in both testicular cell nucleus and cytoplasm, localized in primary spermatocyte nucleus and evident in sertoli cell cytoplasm.

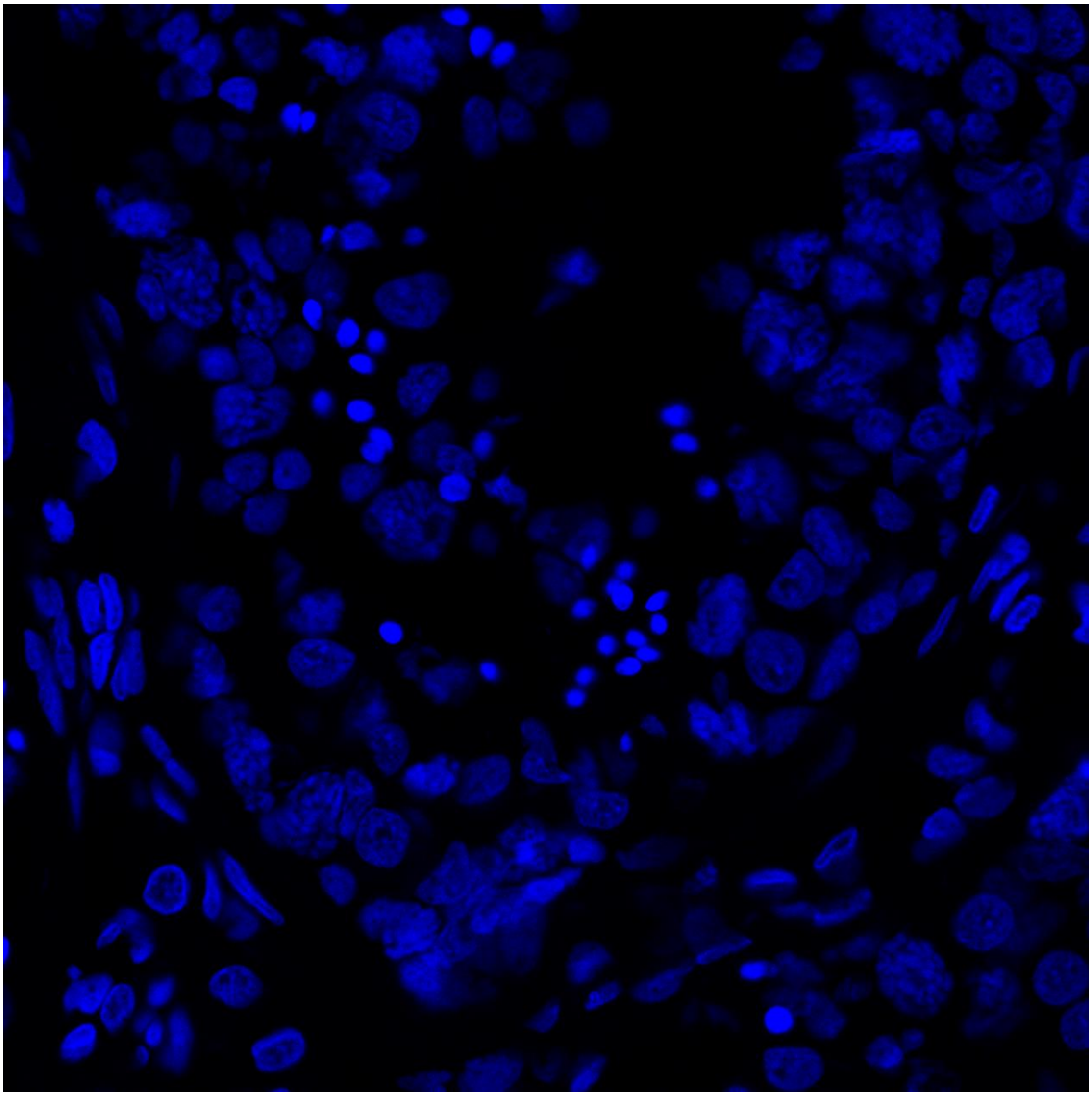

(B) Nuclei are stained with DAPI (blue).

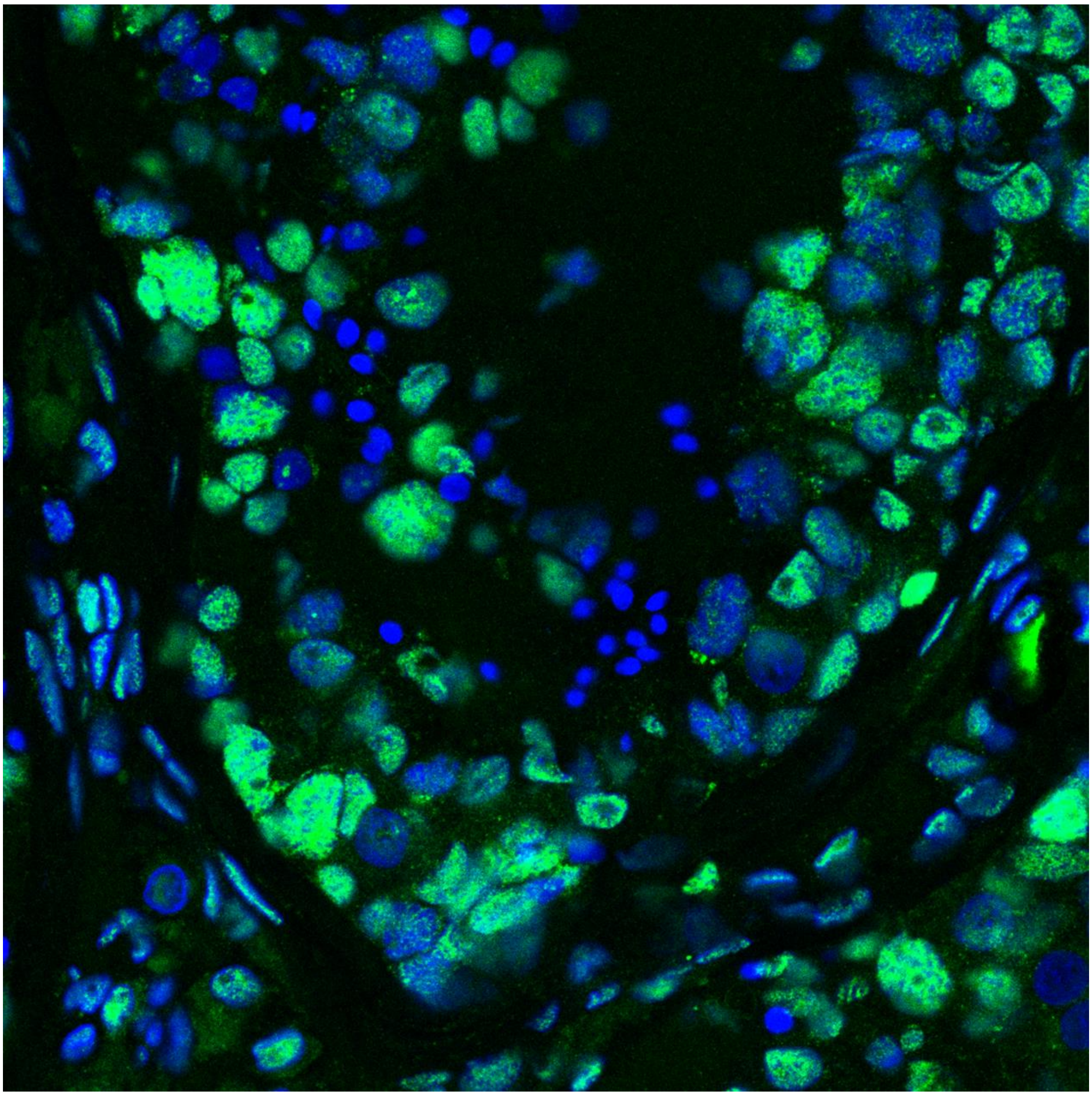

(C) FAM9B (green) is clearly present in both testicular cell nucleus (blue) and cytoplasm, localized in primary spermatocyte nucleus, and evident in sertoli cell cytoplasm. Bars = 10 μm.
