# Supplementary material for: FAM9B serves as a novel meiosis-related protein localized in meiotic chromosome cores and is associated with human gametogenesis
Source: PLoS One. 2021 Sep 10;16(9):e0257248. doi: 10.1371/journal.pone.0257248 (PMC8432983; doi:10.1371/journal.pone.0257248)
Supplement: S3 Raw images — (PDF) [file pone.0257248.s003.pdf]

Immunostaining results for FAM9B expression in human ovarian sections.

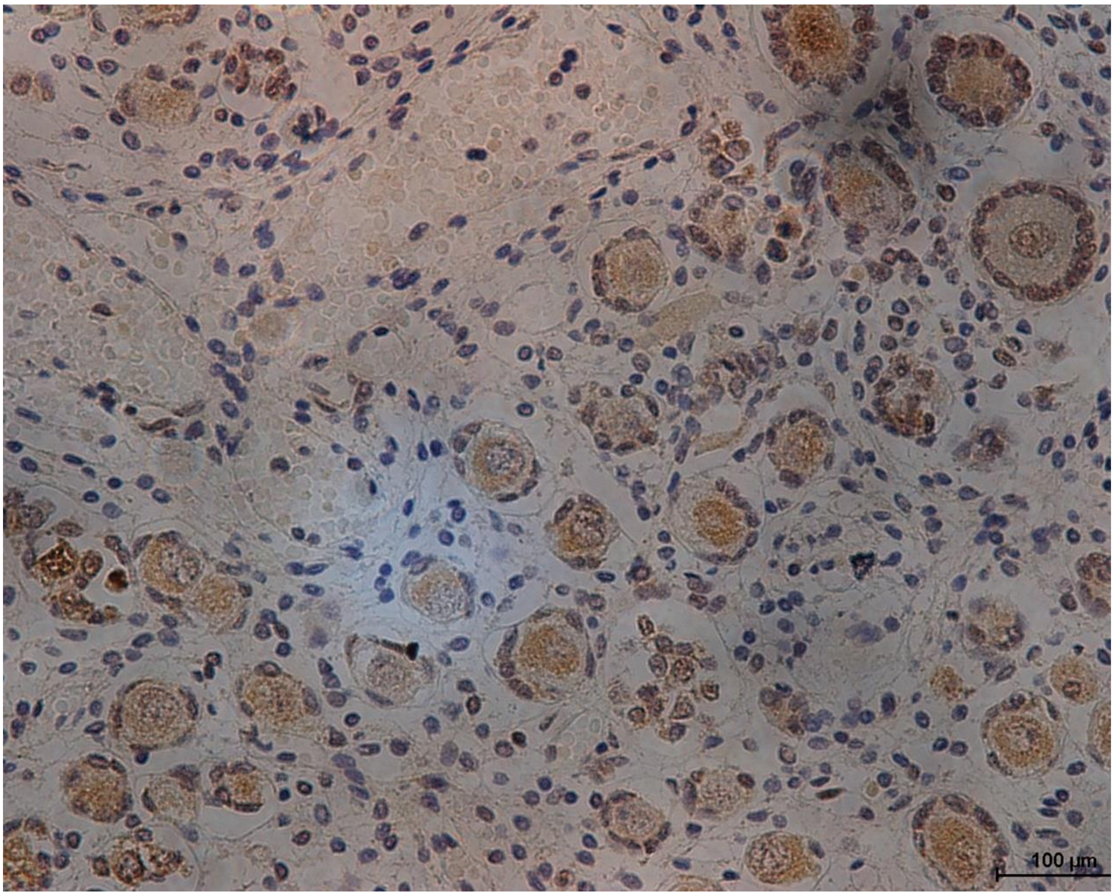

(A) In ovary cells, FAM9B was localized in the cytoplasm of germ cells.

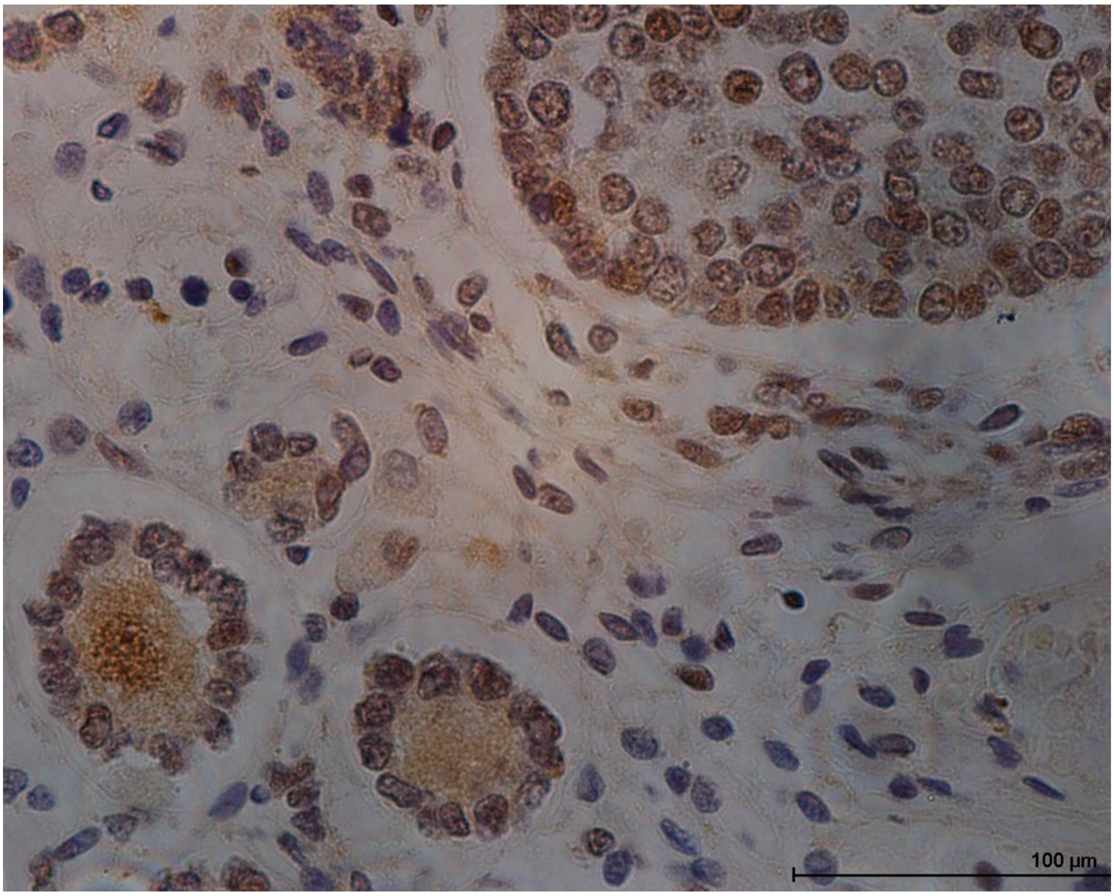

(B) In ovary cells, FAM9B was localized in the nucleus and cytoplasm. FAM9B is clearly present in ovarian sections.

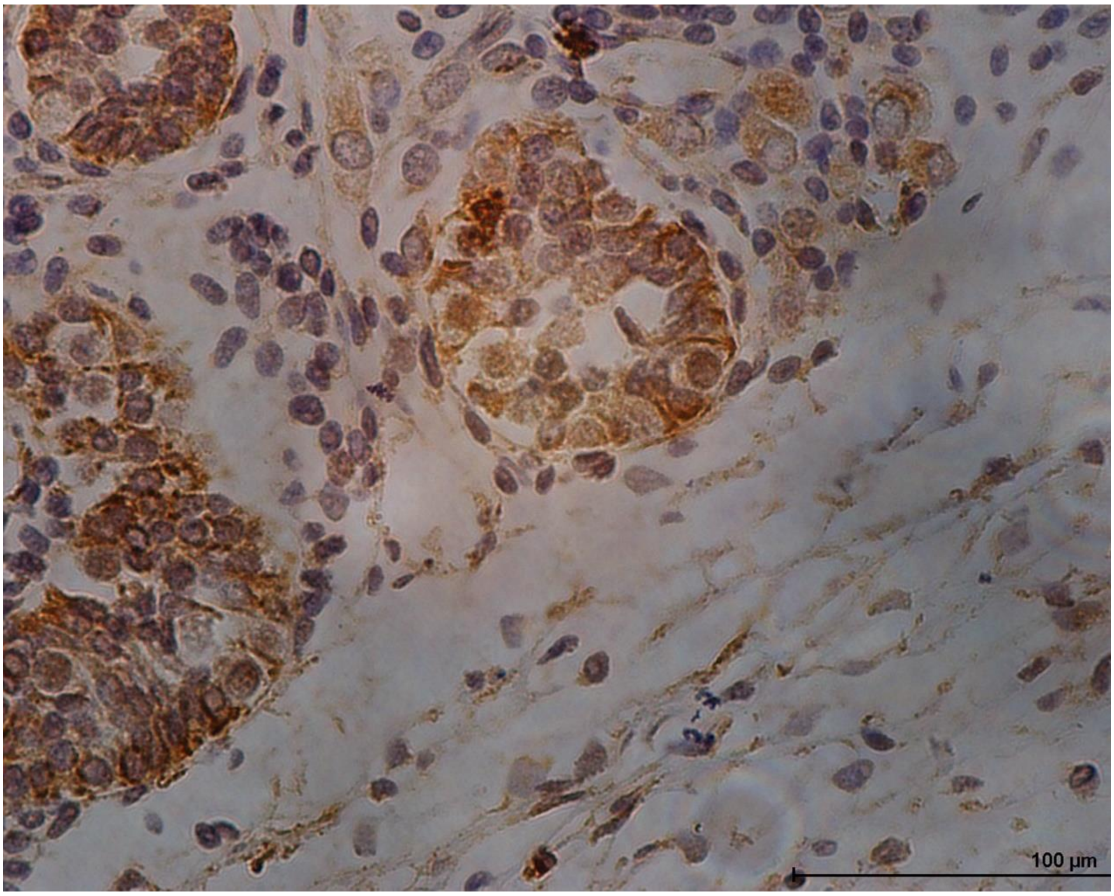

(C) FAM9B is distributed in adult ovaries and localized in granulosa cells.

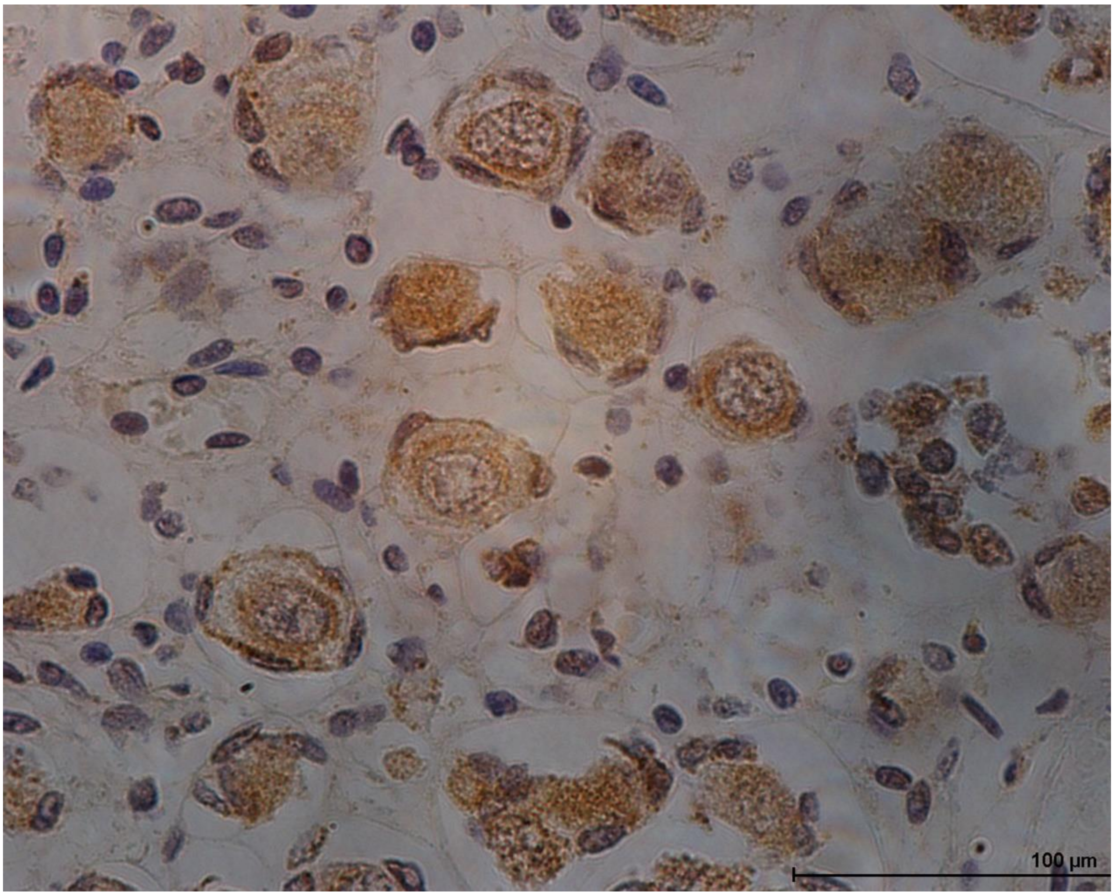

(D)FAM9B is also evident in follicle cell nucleus and diffusely dispersed in granular cell cytoplasm. Bars = 10 µm.
