# Supplementary material for: FAM9B serves as a novel meiosis-related protein localized in meiotic chromosome cores and is associated with human gametogenesis
Source: PLoS One. 2021 Sep 10;16(9):e0257248. doi: 10.1371/journal.pone.0257248 (PMC8432983; doi:10.1371/journal.pone.0257248)
Supplement: S4 Raw images — (PDF) [file pone.0257248.s004.pdf]

FAM9B and SYCP3 proteins are partly co-localized in chromosome sections.

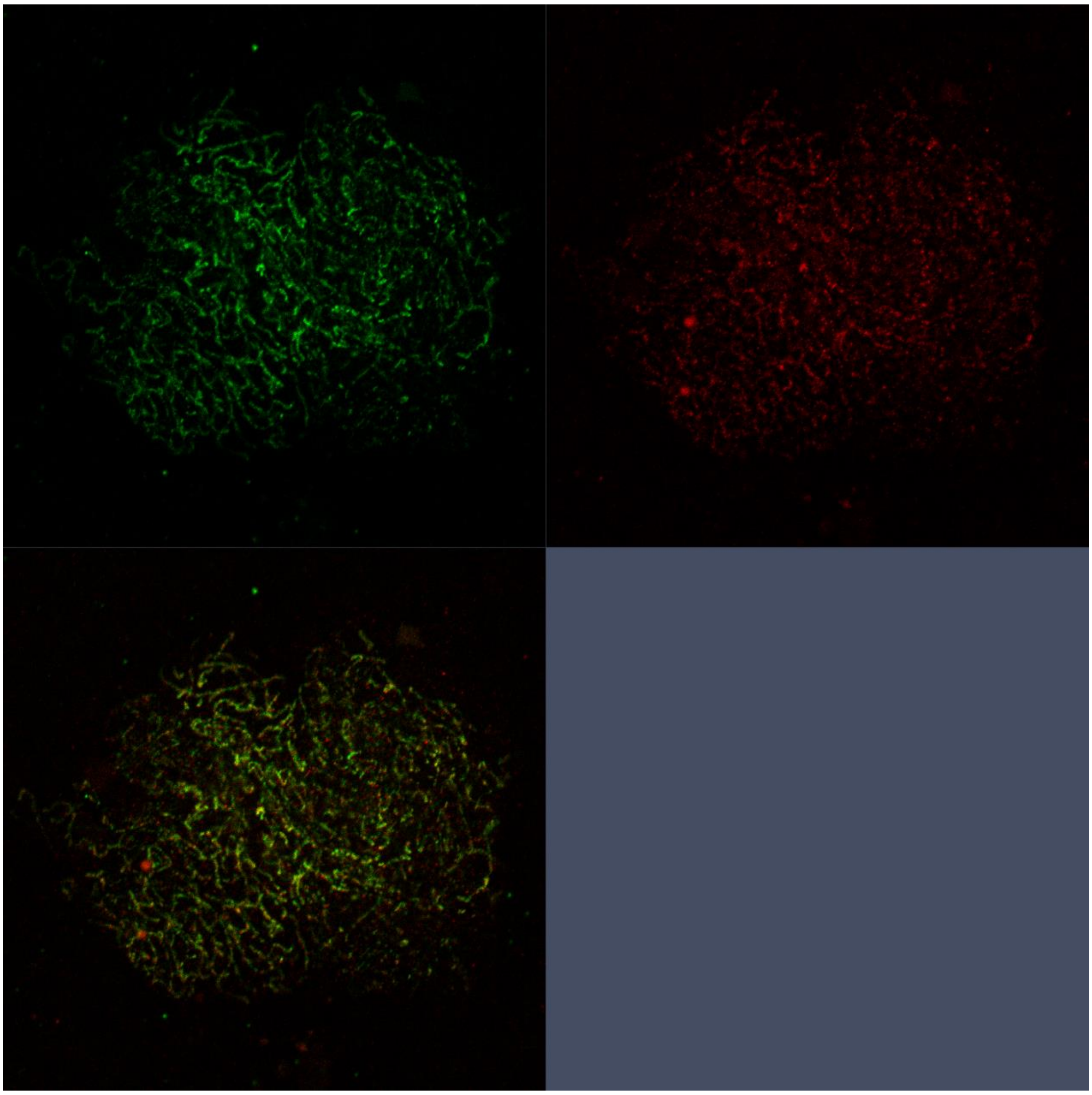

(A). FAM9B (green) and SYCP3 (red) are co-localized in SC of chromosome sections in the leptotene of primary spermatocytes.

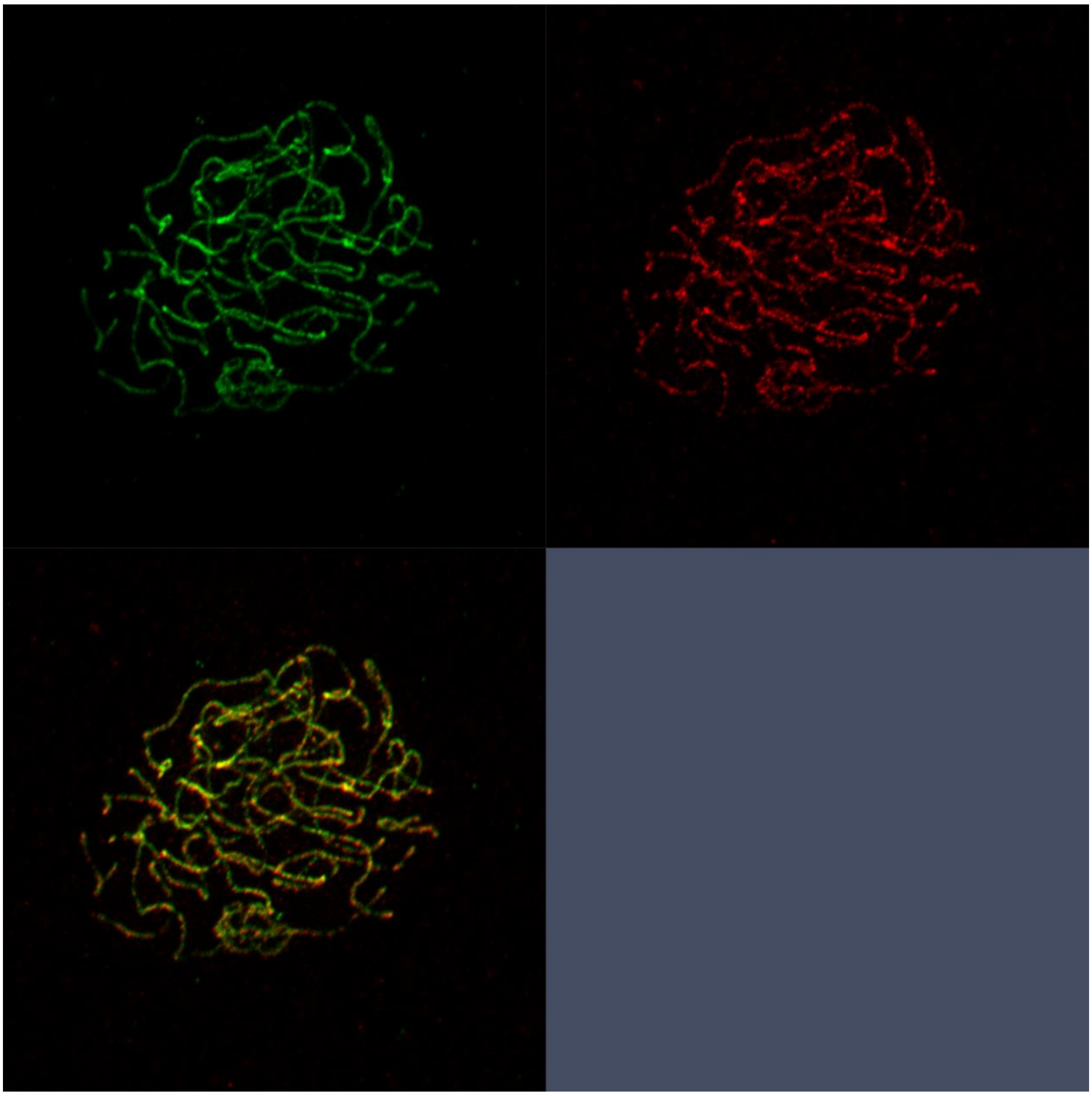

(B). FAM9B (green) and SYCP3 (red) are co-localized in SC of chromosome sections in the zygotene of primary spermatocytes.

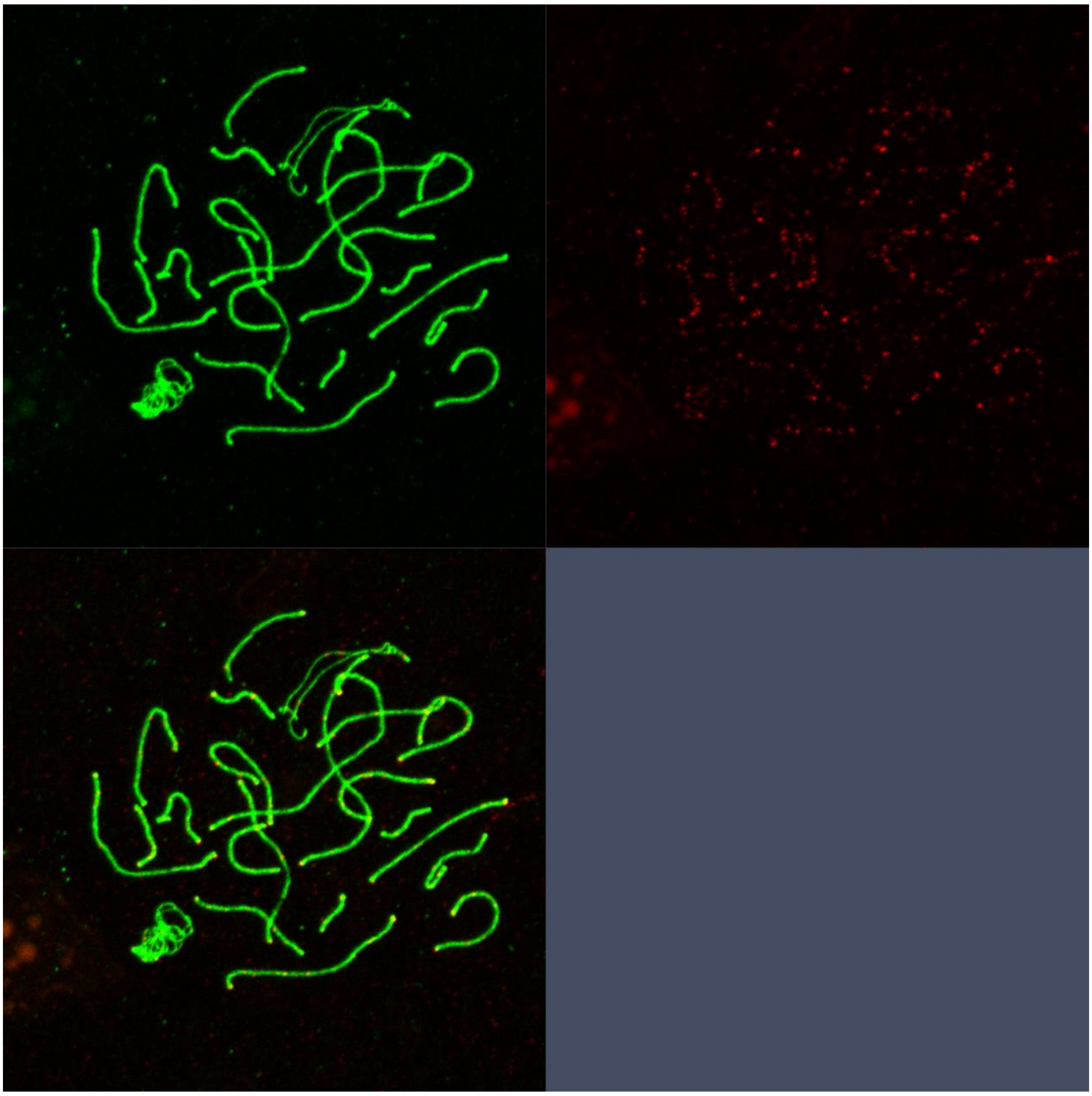

(C). FAM9B (green) and SYCP3 (red) are co-localized in SC of chromosome sections in the pachytene of primary spermatocytes.

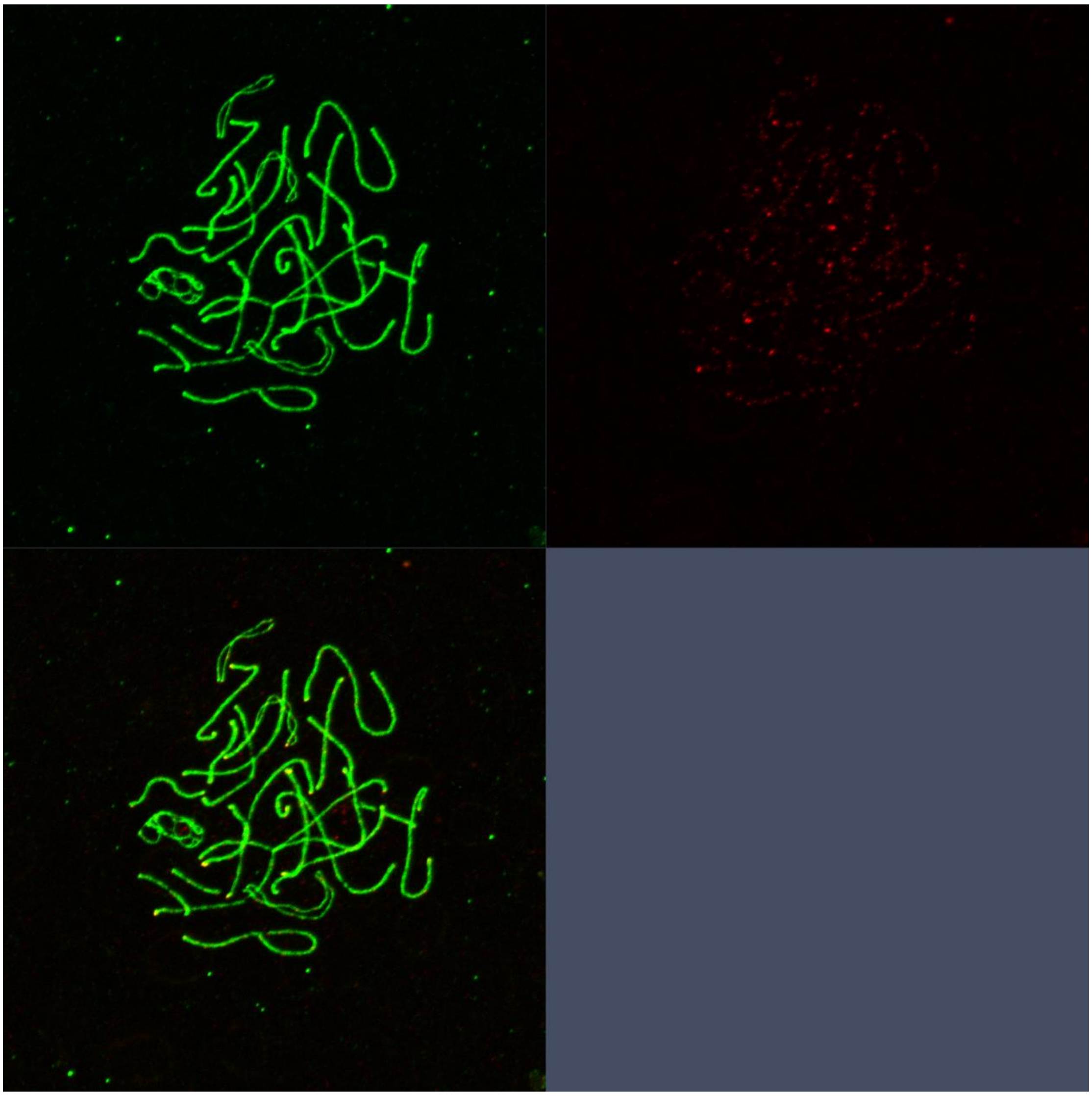

(D). FAM9B (green) and SYCP3 (red) are co-localized in SC of chromosome sections in the early diplotene of primary spermatocytes.The pictures are representative of three independent experiments. Scale bar = 5 µm.
