# Supplementary material for: FAM9B serves as a novel meiosis-related protein localized in meiotic chromosome cores and is associated with human gametogenesis
Source: PLoS One. 2021 Sep 10;16(9):e0257248. doi: 10.1371/journal.pone.0257248 (PMC8432983; doi:10.1371/journal.pone.0257248)
Supplement: S5 Raw images — (PDF) [file pone.0257248.s005.pdf]

FAM9B and γH2AX proteins are partly co-localized in spermatocytes.

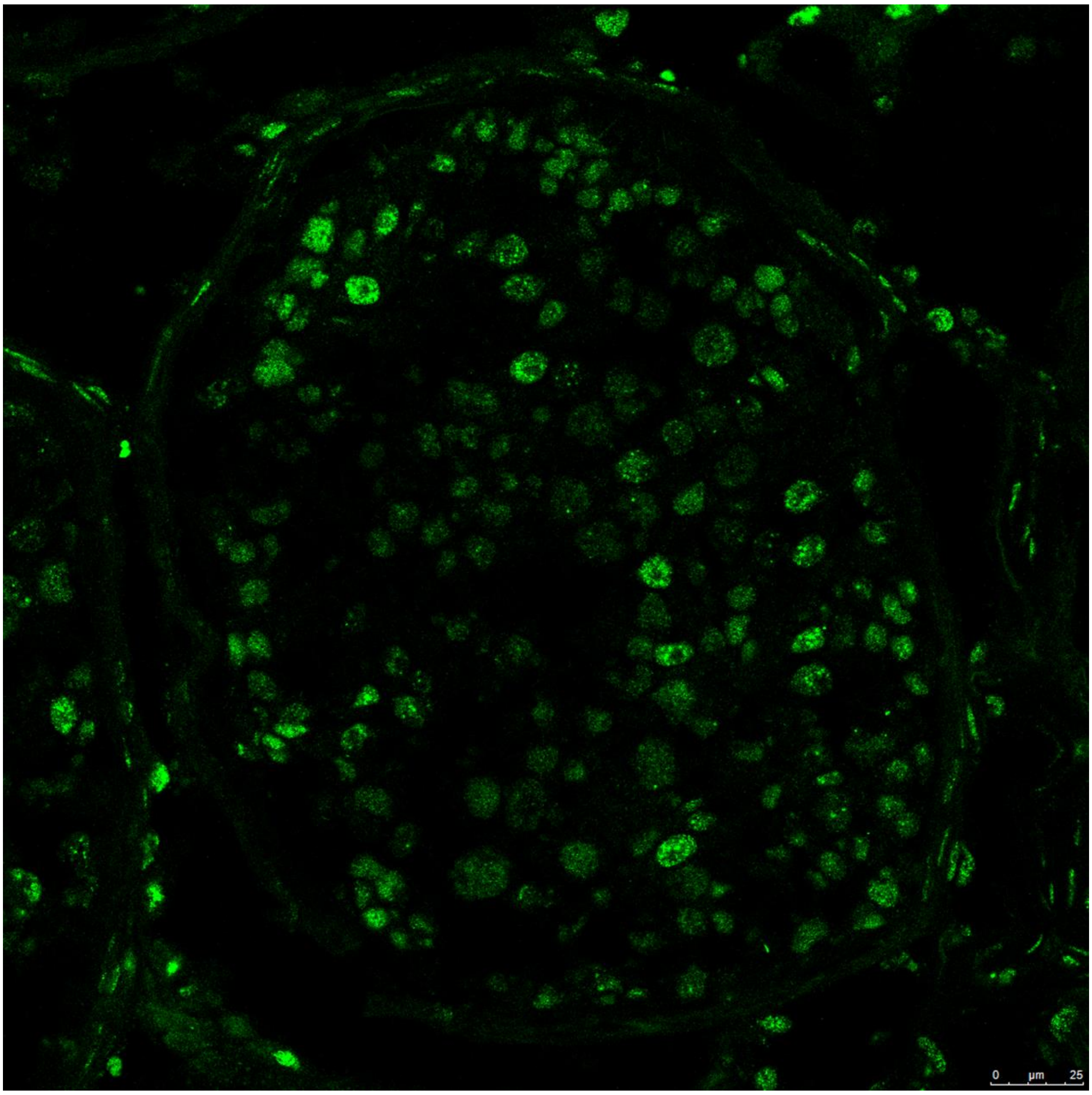

(A) FAM9B (green) localized in the nuclear of spermatocytes.

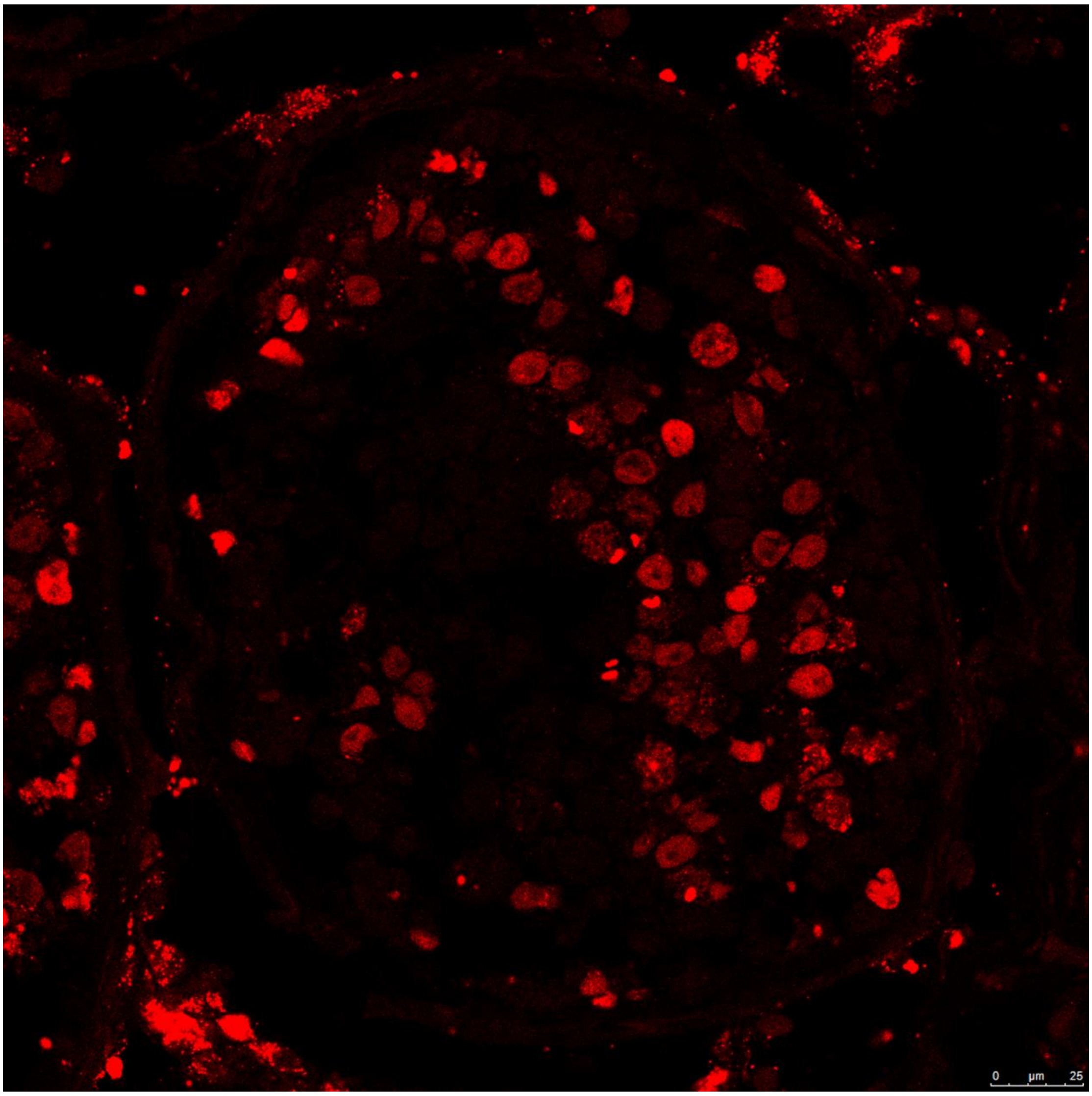

(B) γH2AX (red) localized in the nuclear of spermatocytes.

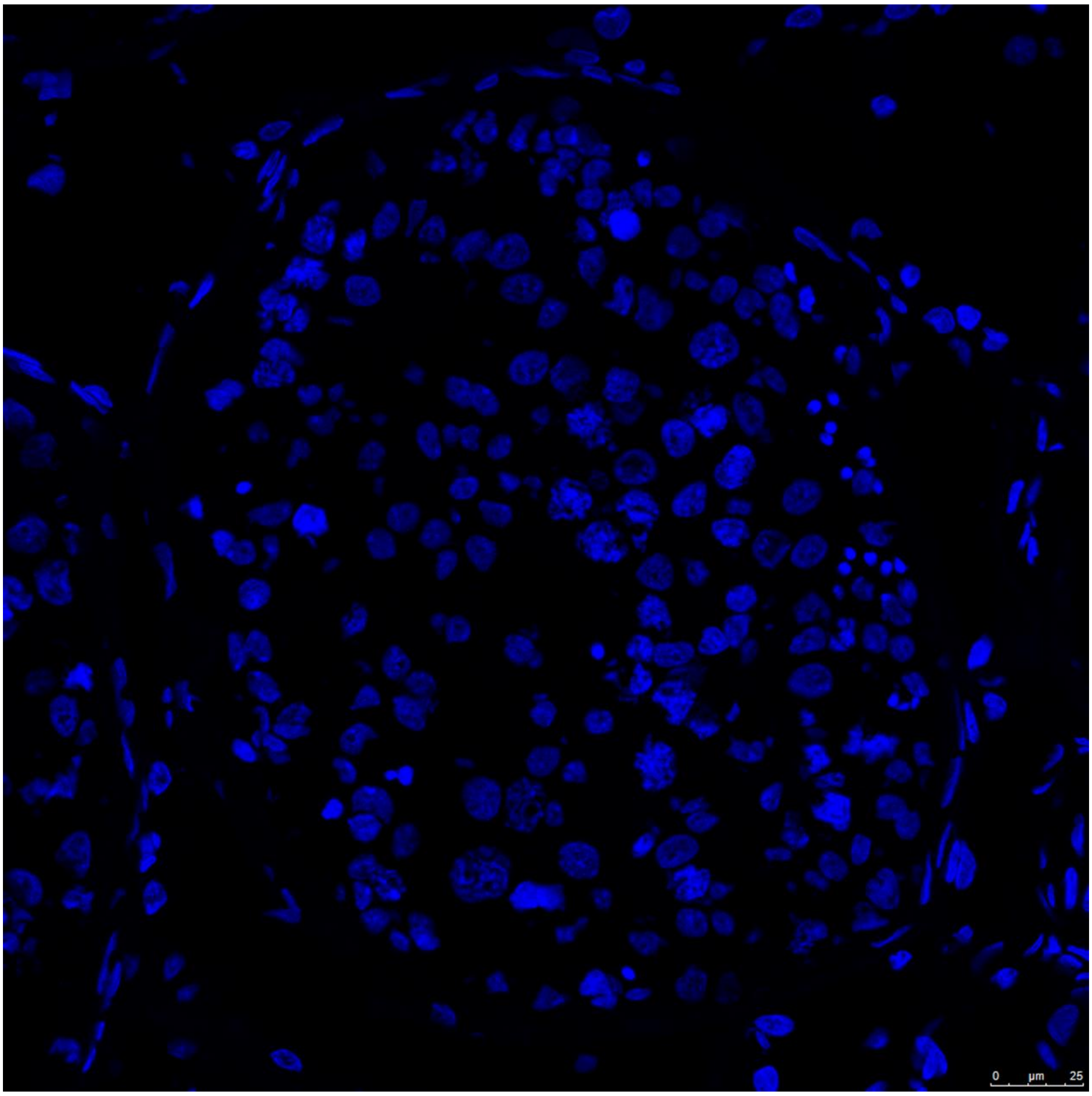

(C) Nuclei are stained with DAPI (blue).

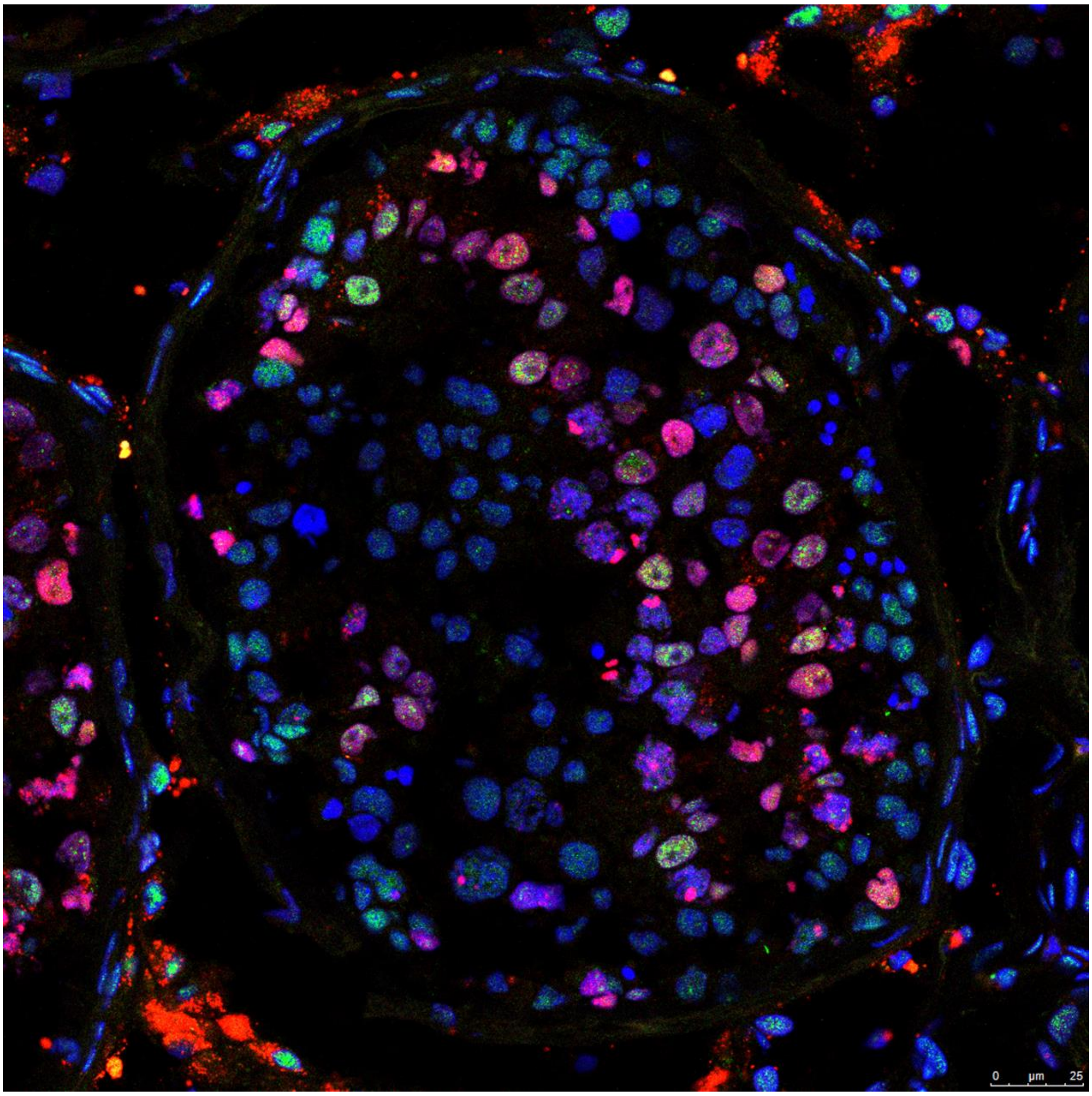

(D) FAM9B (green) and γH2AX (red) are co-localized in the nuclear of spermatocytes. They have a similar distribution pattern. Scale bar = 10 μm.
