# Supplementary material for: FAM9B serves as a novel meiosis-related protein localized in meiotic chromosome cores and is associated with human gametogenesis
Source: PLoS One. 2021 Sep 10;16(9):e0257248. doi: 10.1371/journal.pone.0257248 (PMC8432983; doi:10.1371/journal.pone.0257248)
Supplement: Attachment — Submitted filename: Response to the reviewers_Liu.doc [file pone.0257248.s006.doc]

Dear Dr. Xuejiang Guo,

We are pleased to let you know that we have finished revision of the manuscript according to the suggestions by you and the reviewers. The point-by-point responses are as the following. Please let me know if any further improvement is needed. Thank you very much for your kind help!

Sincerely yours,

Dr. Ping Liu

Center for Reproductive Medicine, Department of Obstetrics and Gynecology,

Key Laboratory of Assisted Reproduction,Ministry of Education,

Peking University Third Hospital; Beijing 100191, PR China

E-mail: Bysylp@sina.com

Reviewers' comments:

Reviewer #1:

1、 Is the manuscript technically sound, and do the data support the conclusion？
This manuscript focused on human gametogenesis which is a highly orchestrated process and involves numerous genes. The authors studied Fam9b, which shows high homology with the SC protein SYCP3. The RT-PCR and western blot results showed Fam9b was expressed in Human testis and ovary at mRNA and protein level. While the IM results revealed the distribution pattern of Fam9b during spermatogenesis and oogenesis. Especially, FAM9B partly co-localized with SYCP3 and γH2AX which indicates the potential function of FAM9B in SC formation and DNA recombination. The design of the article is reasonable and the results can support the conclusion.But some details should be added, such as the replication of the experiments, the negative control of Fig2B. And the information of γH2AX should be added in Introducton part since it is very important for the conclusion of FAM9B in meiotic recombination.

***Response:*** We thank the reviewer for this suggestion. We have revised the Fig2B according to the reviewer’s suggestion. And as reviewer suggested that we have modified the introduction section and added the information of γH2AX in meiotic recombination. Such as “Chromatin reorganization requires formation and repair of DNA double-strand breaks (DSBs). DSB generation rapidly results in the phosphorylation of histone H2A variant H2AX . Because H2AX phosphorylation (γ-H2AX) is abundant, fast, and correlates well with each DSB, it is the most sensitive marker that can be used to examine the DNA damage produced and subsequent repair of the DNA lesion . γ-H2AX appeared to be located in unsynapsed chromosomal segments during male meiotic prophase I. Several waves of H2AX phosphorylation/dephosphorylation coupled to various developmental phases of spermatogonia and spermatocytes, as well as to spermatid differentiation. Previously, we found that SLX2-conserved Cor1 domain was highly homologous with SYCP3. Its co-localization with γH2AX and interaction with TIP60 suggested that SLX2 might be involved in DNA recombination and DSBs during meiosis.” Please check them in the tracked version of the MS.

2、 Has the statistical analysis been performed appropriately and rigorously?
The qPCR was carried out to study the mRNA level of FAM9B in different tissues which showed that FAM9B was highly expressed in reproductive organs. The figure legends should include the information on sample type, replication and so on.

***Response:*** It is a very constructive suggestion. We have modified the information of figure legends. All results were repeated for three times.

3、 Have the authors made all data underlying the findings in their manuscript fully available?
The data showed in this manuscript is complete and could support the results. But in the figure legends, the descriptive information should be added such as the sample type, the replication, the color and arrows information.

***Response:*** As reviewer suggested that we have modified the information of figure legends. Please check them in the tracked version of the MS.

4、 Is the manuscript presented in an intelligible fashion and written in standard English?
The article is well written and easy to understand, but there are still some sentences that need to be revised. Such as:
LINE19: has been found
Line 20: delete” the ”
Line21: reported previously
Line25: co-localized
Line28: indicated
Line 98: samples
Line 111: blocked in TBST ?
LINE 145: Fam9b may have functions as SYCP3 in spermatogenesis.
Line161: expression---distribution or localization ?
Line 170: meiosis
Line 183: co-localization?? Or only to explore the localization pattern of Fam9b and γH2AX.

# *Response:* We appreciate it very much for the reviewer’s helpful suggestions. As the reviewer suggested, we re-check and modified the symbols or characters. And the modified manuscript was re-edited by International Science Editing. Please check them in the tracked version of the MS.

Reviewer #2: In this manuscript, Zhuang et al. explored the expression pattern and localization of a novel protein FAM9B that may play a role in meiosis. It is interesting to note that this protein has a similar distribution pattern to the localization of Sycp3 and γH2AX. Overall, this work showed good data quality and revealed FAM9B may offer a new perspective for understanding the molecular mechanisms involved in meiosis of human gametogenesis.It is better to further analyze the co-localization of FAM9B and γH2AX on spermatocyte spreading slides, to provide more accurate evidence for whether FAM9B is a bio-marker of DSB.Spelling and grammatical checking is needed before publication.

***Response:*** We thank the reviewer for this suggestion. We have completely revised the manuscript and given the full names for all the acronyms. As the reviewer suggested, we re-check and modified the symbols or characters. We have made the correction of the English expression and the modified manuscript was re-edited by International Science Editing.

And we have modified the introduction section and added the information of γH2AX and FAM9B in meiotic recombination and co-localization. Such as “Chromatin reorganization requires formation and repair of DNA double-strand breaks (DSBs). DSB generation rapidly results in the phosphorylation of histone H2A variant H2AX . Because H2AX phosphorylation (γ-H2AX) is abundant, fast, and correlates well with each DSB, it is the most sensitive marker that can be used to examine the DNA damage produced and subsequent repair of the DNA lesion . γ-H2AX appeared to be located in unsynapsed chromosomal segments during male meiotic prophase I. Several waves of H2AX phosphorylation/dephosphorylation coupled to various developmental phases of spermatogonia and spermatocytes, as well as to spermatid differentiation. Previously, we found that SLX2-conserved Cor1 domain was highly homologous with SYCP3. Its co-localization with γH2AX and interaction with TIP60 suggested that SLX2 might be involved in DNA recombination and DSBs during meiosis.” Please check them in the tracked version of the MS.
